# Sexual function after radical cystectomy in males with bladder carcinoma: a six-year longitudinal single-centre study

**DOI:** 10.3389/fruro.2023.1100516

**Published:** 2023-10-03

**Authors:** Claudia E. Pronk, Leonore F. Albers, Lothar D. J. Kuijper, Kees Hendricksen, Melianthe P. J. Nicolai

**Affiliations:** ^1^ Urology Department, The Netherlands Cancer Institute-Antoni van Leeuwenhoek Hospital, Amsterdam, Netherlands; ^2^ Department of Urology, Leiden University Medical Centre, Leiden, The Netherlands; Department of Urology, Antoni van Leeuwenhoek Hospital, The Netherlands Cancer Institute, Amsterdam, Netherlands; ^3^ Department of Health Science, Faculty of Science, Amsterdam Public Health Research Institute, Vrije Universiteit Amsterdam, Amsterdam, Netherlands

**Keywords:** bladder cancer, sexual function, radical cystectomy, male, erectile function

## Abstract

**Introduction:**

Little is known about the long-term effects on sexual function (SF) after radical cystectomy (RC) in bladder carcinoma (BC) patients.

**Aim:**

To assess the course of SF in BC patients who underwent RC, and individual characteristics that influence the sexual outcome during a six-year follow-up.

**Methods:**

In this longitudinal study, 62 BC patients treated with RC were included between 2008 and 2022. Patients filled out validated questionnaires as part of the regular care at baseline, three months, six months, 12 months and thereafter yearly post RC.

**Outcome measures:**

To determine the sexual function, the International Index of Erectile Function questionnaire was filled in and a linear mixed model for repeated measures was conducted. The analysed clinical variables included sexual preserving cystectomy (SPC), age, type of diversion, treatment, comorbidities, tumour status, smoking habits, patient status and open- or robot-assisted RC. A Generalised Linear Mixed Model was used to evaluate the impact on Quality of Life with the QLQ-C30 questionnaire.

**Results:**

After an initial decrease of sexual function post-RC, no change in SF was seen during the six years of follow-up. A statistically significant difference in sexual health was found between SPC and standard RC (p=0.015), which was time-dependent. Patients with an orthotopic ileal neobladder experienced a significantly better SF than those with a Bricker’s ileal conduit (p=<0.001). A younger age also seemed to yield beneficial outcomes regarding SF (p=0.004). Other analysed clinical variables did not influence the course of SF. A statistically significant positive correlation was found between Quality of Life- and SF scores (p=0.004). Robot-assisted RC resulted in higher Global Health scores than open RC (p=0.001).

**Conclusions:**

RC has a severe impact on sexual function. Although SPC, collinear with the use of an orthotopic ileal neobladder and younger patient age show better outcomes in SF, erectile dysfunction post-RC is moderate to severe in the vast majority of patients.

## Introduction

The European Urology Association (EAU) Guidelines recommends radical cystectomy (RC) and urinary diversion for patients with muscle-invasive bladder carcinoma (MIBC) and treatment-unresponsive or recurrent non-muscle invasive bladder carcinoma (NMIBC) (1). Quality of Life (QoL) is an important issue for patients who survive bladder cancer (BC) and RC (2, 3). BC patients experience significant declines in physical, mental, and social health-related quality of life (HRQoL), where sexual function (SF) is an important aspect of QoL in cancer patients, especially in younger patients (4, 5).

RC can be performed either as an open radical cystectomy (ORC) or as a robot-assisted radical cystectomy (RARC), where additionally a sexual function-preserving technique can be conducted. Research suggests that sexual function-preserving cystectomy (SPC) may result in better sexual outcomes than standard cystectomy, without compromising oncological outcomes when carefully selected (6). Although the type of urinary diversion does not affect the oncological outcome, ileal orthotopic neobladders show superiority regarding SF over Bricker’s ileal conduit diversion (1, 7).

Sexual dysfunction after RC is common and can be a result of iatrogenic nerve damage due to surgery, as well as post-operative body image changes due to the urinary diversion (2). In men, erectile dysfunction (ED) is the most common sexual side effect after RC and wide ranges of ED rates have been reported (8). In addition to ED, other sexual changes that might occur include loss of penile length, reduced sexual desire and orgasmic dysfunction including painful orgasm and climacturia (9). Factors that affect the sexual outcomes after RC are the patient’s age, comorbidities, BMI, ASA-score, use of penile rehabilitation tools or programs, tumor status, chemotherapy, smoking habits, and preoperative erectile function (8, 10–14).

Although sexual dysfunction is common after RC, it is frequently overlooked by healthcare professionals (15). Little is known about the extent of SF and its course after RC in BC patients and the individual characteristics and factors that may influence the recovery of SF. Therefore, personalized information about the risk of sexual dysfunction after RC for patients is limited. The aim of this study is to evaluate the course of the SF, including sexual desire and satisfaction, and to identify factors that influence the recovery of SF in male BC patients after RC.

## Methods

### Patients

This cohort included male BC patients with RC, who were treated at The Netherlands Cancer Institute-Antoni van Leeuwenhoek Hospital between 2008 and 2022. The patients were operated with either ORC or RARC, where additionally a SPC could have been performed. To perform a urinary deviation the intracorporeal approach was used.

Patients could be considered for a sexuality-sparing cystectomy (SPC) if they had organ-confined disease without the presence of tumor at the level of the prostate, urethra, or bladder neck. During SPC, the entire prostate, including seminal vesicles, vas deferens, and neurovascular bundles, are preserved. Alternatively, only the neurovascular bundles are left in place. The RC was performed by one of eight fellowship-trained urologic oncologists, who aim to perform >20 RC’s per year.

### Questionnaires

During the first visit in the hospital, patients were asked to give permission to receive questionnaires and to use the anonymized data for research. Filling out validated questionnaires is part of the regular care in the Netherlands Cancer Institute at baseline, three months, six months, 12 months and thereafter yearly post RC. To determine the SF and QoL, the three administered self-reported questionnaires that were used were: 1) the International Index of Erectile Function 15 (IIEF-15) and 2) the European Organization for Research and Treatment of Cancer (EORTC) core quality of life questionnaire (QLQ-C30) (16, 17). The questionnaires were provided via email or on paper and were filled out in the privacy of the patients’ home.

Patients who did not fill in a questionnaire before surgery (at baseline) and at least one follow-up moment after surgery were excluded. Patients who filled in the questionnaire more than two years pre-RC were excluded, as it was deemed that the SF and global QoL was not representative more than two years pre-RC. Moreover, the questionnaires that were filled in within the first 30 days after the RC, were excluded, since patients are expected to still be recovering from their surgery.

The IIEF-15 questionnaire is a validated specific questionnaire regarding the SF of men and contains five domains (16). Calculation of the scores was done according to the manual of Rosen et al., where a higher score correlates with a higher SF (16). The answer ‘does not apply’ was recorded as a missing value. If a patient filled in two answers for one question, this was also reported as a missing value. No exclusion of the non-sexually active population was executed since this would give a better representation of the population that visits the urologist’s consultation room. Exclusion might have led to an overestimation of the SF.

Due to the rather high number of missing values in the questionnaire, exploration was performed. In this process, means are calculated rather than the sum score of the questions, to reduce underestimation of the SF. This was performed for both the total scores and the individual five domains. A mean IIEF-score of >4 is considered a good SF-score for sexual intercourse.

The EORTC QLQ-C30 has been validated and incorporates multi-item scales regarding functional and symptom scales, and a Global Health Status/QoL (gHS/QoL) scale (18). The scores on QoL were calculated, and the missing values were corrected for in accordance with the EORTC QLQ-C30 Scoring Manual (17).

### Statistical analysis

To convert the data from wide to long format, STATA 15 for Windows (StatCorp, College Station, T.X., USA) was used. All statistical analyses were conducted using IBM SPSS Statistics 27 for Windows (IBM corp., Armonk, N.Y., USA). For the descriptive analyses, the means with standard deviations (SD) were presented for continuous outcomes, and categorical variables were presented as a number with a percentage. Independent Samples T-Test was used to compare means, and a two-sided *p*-value was reported based on assumption of equal or unequal variances. A two-sided Fisher’s exact test was performed to compare dichotomous variables.

For the analyses, dichotomous variables were computed to simplify the analyses and increase the power of the analyses for the variables SPC (prostate-, and nerve-sparing procedures were recoded into ‘any type of sexual preserving’) and type of therapy (brachy-, external beam radio-, neo-adjuvant chemo- and immune-therapy were recoded into ‘any type of therapy’). For the variable ‘comorbidities’, a sum of the comorbidities was computed to also simplify the analysis.

A Linear Mixed Model analysis for repeated measures was performed to obtain the course of the SF and the influence of different variables on this course. Since it has been shown that a linear mixed model can better correct for missing values than multiple imputation, only the mixed model was performed (19). For the Linear Mixed Model analyses, the Repeated Covariance Type structure was chosen by performing the crude analyses with several covariance types (i.e. Diagonal, Auto-Regressive 1 Heterogeneous and Unstructured) and choosing the covariance type that had the lowest Akaike Information Criterium (AIC). A random intercept for the patients was performed (for adjustment of multiplicity), with an Unstructured covariance type for the random effect. Covariates could not be included integrally to the fixed effects of the model due to the low power of the analyses and were thus evaluated separately. It was determined if a variable had an influence on the course of the SF by evaluating the *p*-value and a change in estimates. If possible, an interaction term of the interested variable with the different time points was added when a significant effect was seen in the model, to determine if the variable is time-dependent. To establish if a model fits the data better when a variable is added, the likelihood ratio chi-squared selection strategy was used (20). For the analysis of the QoL from the EORTC QLQ-C30 questionnaire, a Generalized Linear Mixed Model (GLMM) was performed. A gamma regression was used for the target variable, the time-points were added as fixed effects and the subject id was added as a random effect with intercept. A *p*-value of <0.05 is deemed as statistically significant.

### Ethical approval

This study followed the principles of the Helsinki Declaration. Institutional review board (number IRBd20-070) approval of the Netherlands Cancer Institute – Antoni van Leeuwenhoek Hospital was obtained.

## Results

### Patients

From September 2008 until February 2022, 62 BC patients were included for analysis. **Table 1** presents their demographic and clinical characteristics, stratified for patients with and without SPC. No variables of the descriptive statistics showed a difference between the two groups, except for age. The group of patients with standard RC had a mean age of 68.1 years, whereas the group with SPC had a mean age of 61.0 years (p=0.005). Furthermore, a statistically significant difference (*p*=<0.001) was found regarding the age between the group with Bricker’s ileal conduit (mean age 68.44 with SD: 6.44) and the group with orthotopic ileal neobladder (mean age 56.50 with SD: 9.27).

**Table 1:** Patient and clinical characteristics.

*Characteristic*	Patients n = 62 (%)	Patients without SPC n = 50 (%)	Patients with SPC n = 12 (%)	*p*-value
***Age (years) at RC*** *Mean (SD)* *Range*	66.7 (8.1) 43-84	68.1 (7.3) 45-84	61.0 (9.1) 43-74	0.005
***Type of RC*** *ORC* *RARC*	38 (61) 24 (39)	32 (64) 18 (36)	6 (50) 6 (50)	0.511
***Type of SPC*** *No SPC* *Prostate-sparing* *Nerve-sparing*	50 (80) 6 (10) 6 (10)	50 (100) 0 (0) 0 (0)	0 (0) 6 (10) 6 (10)	
***Type of Urinary Diversion*** *Bricker’s Ileal Conduit* *Orthotopic Ileal Neobladder* *Continent Cutaneous Diversion*	54 (87) 6 (10) 2 (3)	46 (92) 2 (4) 2 (4)	8 (67) 4 (33) 0 (0)	
Type of Therapy* *No Adjuvant Therapy* Brachytherapy** External Beam Radiotherapy ** *Neo-adjuvant Chemotherapy* *Neo-adjuvant Immunotherapy*	27 (44) 4 (6) 7 (11) 27 (44) 3 (5)	22 (44) 2 (4) 6 (12) 23 (46) 2 (4)	5 (42) 2 (17) 1 (8) 4 (33) 1 (8)	
***Smoking Habits*** *User* *Non-user* *Unkown*	40 (68) 19 (32) 3	32 (68) 15 (32) 3	8 (67) 4 (33)	1.000
***Body Mass Index (kg/m^2^)*** *Mean (SD)* *Range* *Missing*	27.0 (3.7) 20.8-33.8 35	27.5 (3.7) 20.8-33.8 28	24.6 (2.9) 21.2-24.6 7	0.107
***ASA-score at RC*** *1* *2* *3* *Missing*	15 (29) 30 (59) 12 (12) 12	11 (28) 23 (59) 5 (13) 11	4 (36) 6 (55) 1 (9) 1	
Comorbidities* *No comorbidities* *Diabetes Mellitus* *Hypertension* *Cardiovascular Disease* *Other type of Cancer* *Other comorbidities*	20 (32) 6 (10) 14 (23) 6 (10) 22 (35) 15 (24)	18 (36) 4 (8) 11 (22) 4 (8) 18 (36) 10 (20)	2 (17) 2 (17) 3 (25) 2 (17) 4 (33) 5 (42)	
***cT*** *a* *is* *1* *2* *3* *4* *Missing*	4 (6) 3 (5) 3 (5) 22 (37) 21 (35) 7 (12) 2	2 (4) 2 (4) 3 (6) 17 (35) 18 (38) 6 (13) 2	2 (17) 1 (8) 0 (0) 5 (42) 3 (25) 1 (8) 0	
***cN*** *0* *1* *2* *3* *Missing*	49 (82) 5 (8) 5 (8) 1 (2) 2	38 (80) 5 (10) 4 (8) 1 (2) 2	11 (92) 0 (0) 1 (8) 0 (0) 0	
***cM*** *0* *1* *Missing*	56 (93) 4 (7) 2	44 (92) 4 (8) 2	11 (100) 0 (0) 0	
***Most Recent Status*** *Free of Disease* *Dead of/with Disease* *Dead of other Disease* *Alive with Disease* *Unknown*	46 (75) 10 (16) 1 (2) 4 (6) 1	37 (74) 8 (16) 1 (2) 4 (8)	9 (82) 2 (18) 0 (0) 0 (0) 1	

ORC, Open Radical Cystectomy; RARC, Robot-Assisted Radical Cystectomy; RC, Radical Cystectomy; SD, standard deviation; SPC, Sexual Preserving Cystectomy.

*Percentages are presented as a percentile of the total amount of patients, as some patients have had multiple treatments or comorbidities, resulting in a sum of percentages that exceeds 100%.

**Brachy- and external beam therapy were performed with curative intent.

The unadjusted SF and HRQoL outcomes of 62 males with BC at baseline, three months, six months, one year and thereafter yearly post-RC are presented in **Tables 2**, **3** respectively. All IIEF-scores are below three, indicating that the men suffered from moderate to severe sexual and erectile dysfunction. Additionally, a difference was found in pre-operative SF between the group without SPC (mean SF of 1.61, SD: 1.25), and a mean SF of 2.91 (SD: 1.45) for the group with SPC (p=0.003).

**Table 2:** Sexual function from the IIEF-questionnaire.

*Time point*	Baseline (pre-RC) Mean ± SD	3 months Mean ± SD	6 months Mean ± SD	1 year Mean ± SD	2 years Mean ± SD	3 years Mean ± SD	4 years Mean ± SD	5 years Mean ± SD	6+ years Mean ± SD
***Cases***	n = 57	n = 33	n = 36	n = 30	n = 23	n = 16	n = 15	n = 7	n = 6
*Total score (0-5)*	1.89 ± 1.39	1.16 ± 0.86	1.34 ± 1.26	1.30 ± 1.09	1.31 ± 1.29	1.02 ± 1.01	1.27 ± 1.25	1.50 ± 1.28	1.93 ± 1.34
*Erectile function (0-5)*	1.77 ± 1.65	0.78 ± 0.93	0.98 ± 1.40	0.92 ± 1.19	1.00 ± 1.40	0.69 ± 1.06	1.07 ± 1.58	1.26 ± 1.46	1.61 ± 1.88
*Orgasmic function (0-5)*	1.89 ± 2.02	0.63 ± 0.79	1.26 ± 1.71	1.18 ± 1.70	1.24 ± 1.75	0.75 ± 1.39	1.07 ± 1.85	1.64 ± 1.82	2.58 ± 2.01
*Sexual desire (1-5)*	2.30 ± 1.18	1.76 ± 1.00	2.16 ± 1.19	2.33 ± 1.14	2.37 ± 1.16	2.00 ± 1.00	2.10 ± 1.04	2.14 ± 1.11	2.42 ± 1.02
*Intercourse satisfaction (0-5)*	1.10 ± 1.49	0.63 ± 1.05	0.80 ± 1.37	0.65 ± 1.23	0.65 ± 1.42	0.46 ± 1.17	0.57 ± 1.32	0.57 ± 1.51	1.39 ± 1.82
*Overall satisfaction (1-5)*	2.92 ± 1.40	2.63 ± 1.52	2.48 ± 1.44	2.59 ± 1.43	2.30 ± 1.38	2.40 ± 1.58	2.71 ± 1.41	2.93 ± 1.70	2.75 ± 1.54

IIEF, International Index of Erectile Function; RC, radical cystectomy SD, standard deviation.

IIEF 5-point Likert scale: 0, did not attempt intercourse; 1, never/almost never; 2, a couple of times; 3, sometimes; 4, often; 5, always/almost always.

**Table 3:** Quality of Life scores from the EORTC-QLQC30 questionnaire.

*Time point*	Baseline (pre-RC) Mean ± SD	3 months Mean ± SD	6 months Mean ± SD	1 year Mean ± SD	2 years Mean ± SD	3 years Mean ± SD	4 years Mean ± SD	5 years Mean ± SD	6+ years Mean ± SD
***Cases***	n = 62	n = 37	n = 36	n = 33	n = 26	n = 19	n = 16	n = 8	n = 9
*Global Health Status*	69.49 ± 21.76	74.78 ± 14.09	75.00 ± 16.67	72.22 ± 22.11	75.96 ± 12.76	72.81 ± 13.56	77.08 ± 18.88	72.9 ± 19.80	65.74 ± 28.09
*Physical Function*	89.97 ± 16.53	84.50 ± 17.78	89.43 ± 12.06	85.63 ± 18.25	88.59 ± 14.611	86.58 ± 11.28	80.00 ± 21.08	85.83 ± 13.07	82.22 ± 19.72
*Role Function*	79.44 ± 26.46	77.03 ± 24.64	85.09 ± 20.79	82.32 ± 27.62	85.90 ± 17.44	80.70 ± 19.45	75.00 ± 25.82	81.25 ± 24.29	70.37 ± 27.36
*Emotional Function*	78.78 ± 19.25	85.81 ± 19.92	86.33 ± 14.92	85.35 ± 17.68	89.10 ± 13.29	86.40 ± 18.88	91.67 ± 12.91	87.50 ± 12.60	82.41 ± 14.70
*Cognitive Function*	90.71 ± 14.12	89.64 ± 13.81	88.89 ± 14.48	87.37 ± 18.64	90.38 ± 14.28	86.84 ± 11.89	88.54 ± 14.55	81.25 ± 18.77	79.63 ± 16.20
*Social Function*	80.87 ± 20.82	84.23 ± 21.50	86.84 ± 19.05	87.37 ± 19.11	87.18 ± 20.17	88.60 ± 18.47	83.33 ± 22.77	81.25 ± 24.39	75.93 ± 25.15
*Fatigue*	27.87 ± 24.96	26.43 ± 20.18	21.37 ± 19.64	22.05 ± 25.17	22.22 ± 18.59	22.81 ± 22.97	23.61 ± 31.13	27.78 ± 27.22	29.63 ± 30.93
*Nausea and Vomiting*	9.84 ± 20.94	2.70 ± 7.36	1.71 ± 6.39	4.04 ± 13.20	1.28 ± 4.53	1.75 ± 5.26	3.13 ± 6.72	0.00 ± 0.00	7.41 ± 16.90
*Pain*	15.57 ± 24.70	13.51 ± 20.73	8.33 ± 14.37	12.62 ± 21.66	7.69 ± 11.77	12.28 ± 16.52	12.50 ± 29.50	4.17 ± 7.72	20.37 ± 33.10
*Dyspnea*	18.89 ± 28.37	17.12 ± 24.37	9.65 ± 18.84	15.15 ± 25.13	11.54 ± 16.17	12.28 ± 19.91	12.50 ± 29.50	4.17 ± 11.79	18.52 ± 29.40
*Insomnia*	22.78 ± 30.99	16.22 ± 23.07	8.55 ± 18.29	16.16 ± 26.51	17.95 ± 21.56	26.32 ± 32.54	14.58 ± 17.08	25.00 ± 38.83	29.63 ± 30.93
*Appetite loss*	16.11 ± 29.75	11.71 ± 25.11	4.27 ± 13.64	8.08 ± 18.69	3.85 ± 14.38	7.02 ± 23.78	6.25 ± 18.13	8.33 ± 23.57	14.81 ± 33.79
*Constipation*	10.00 ± 21.52	12.04 ± 18.09	10.63 ± 19.15	13.54 ± 22.17	7.69 ± 14.32	14.04 ± 25.6	8.33 ± 19.25	4.17 ± 11.79	14. 81 ± 29.40
*Diarrhea*	6.01 ± 14.28	3.60 ± 10.49	6.14 ± 13.10	8.33 ± 18.93	2.56 ± 9.06	3.51 ± 10.51	8.89 ± 15.26	12.5 ± 17.25	3.70 ± 11.11
*Financial difficulties*	22.40 ± 25.62	16.22 ± 24.37	16.67 ± 21.57	16.67 ± 23.95	14.10 ± 21.44	12.28 ± 22.80	20.83 ± 23.96	16.67 ± 25.20	25.93 ± 27.78

QLQ-C30. Quality of Life Questionnaire for Cancer patients (30 questions); SD, standard deviation; RC, radical cystectomy; scores range from 0 to 100.

### Linear mixed model analyses

**Figure 1:**
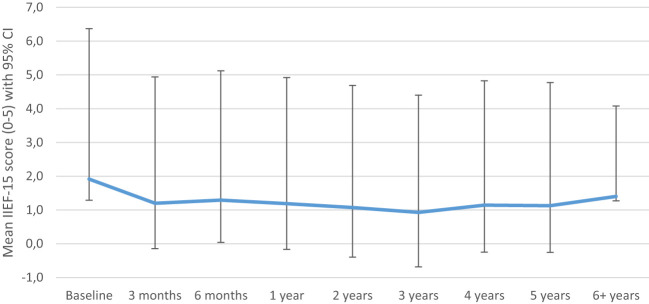
Crude sexual function model (mean IIEF-15 score). IIEF 5-point Likert scale: 0 = did not attempt intercourse; 1 = never/almost never; 2 = a couple of times; 3 = sometimes; 4 = often; 5 = always/almost always.

In **Figure 1**, the unadjusted longitudinal analysis of the mean IIEF-15 total score is depicted for 62 bladder carcinoma patients over a time-period of 6 years (intercept: 1.404, *p*=0.036, Time: *p*=0.012). There was neither an improvement, nor a decrease in SF after the RC (p=0.305). In the **Appendix** the output including -2LL and degrees of freedom from the linear mixed model analyses can be found in **Supplementary Tables 1** until **6**.

**Figure 2:**
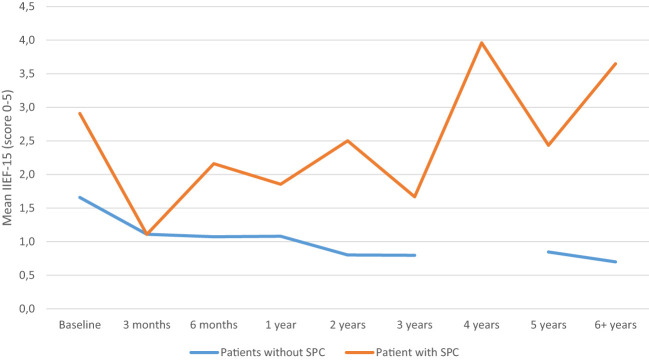
Sexual function model adjusted for sexual preserving cystectomy (SPC). IIEF 5-point Likert scale: 0 = did not attempt intercourse; 1 = never/almost never; 2 = a couple of times; 3 = sometimes; 4 = often; 5 = always/almost always.

The course of SF was influenced by SPC (p=0.015). The course of SF is shown in **Figure 2**; the effect of the SPC was time-dependent (p < 0.05). Furthermore, the mean IIEF-15 score seems to increase one-year post-RC for the patients with SPC.

Age at the time of cystectomy had an influence on the course of SF corrected for SPC (estimate: -0.44, *p*=0.004). Higher age resulted in a lower SF. Analysis with age as an interaction term with the time-points without SPC showed a significant difference (*p*<0.05), indicating an effect of age on the SF over time. At baseline, elderly patients had a decreased SF compared to younger patients (estimate: -0.152, *p*=0.002). However, post-RC, the difference of SF between elderly and younger patients became smaller, showing that even though SF decreased for all patients, the negative effect of surgery in elderly is not as large as in younger patients.

**Figure 3:**
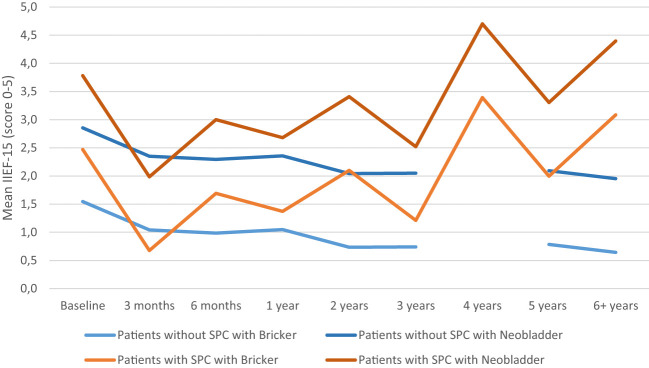
Sexual function model adjusted for sexual preserving cystectomy (SPC) and type of diversions (Bricker’s ileal conduit or orthotopic ileal neobladder). IIEF 5-point Likert scale: 0 = did not attempt intercourse; 1 = never/almost never; 2 = a couple of times; 3 = sometimes; 4 = often; 5 = always/almost always.

The type of diversion performed influenced the course of SF in those with Bricker’s ileal conduit diversion having an estimate of -1.309 compared to orthotopic ileal neobladder, (*p*<0.001). Orthotopic ileal neobladder had better SF scores compared to those with Bricker’s diversion. Addition of this variable showed an improvement of the model (difference -2LL = 23.63 for one degree of freedom). The different courses of SF for patients with and without SPC, with either Bricker’s or orthotopic ileal neobladder is shown in **Figure 3**.

The individual addition of the number of comorbidities, individual comorbidities (diabetes mellitus, hypertension, and any other type of cancer), smoking habits, type of therapy, patient- and tumor status and surgical technique (ORC or RARC) did not influence the course of SF.

**Figure 4:**
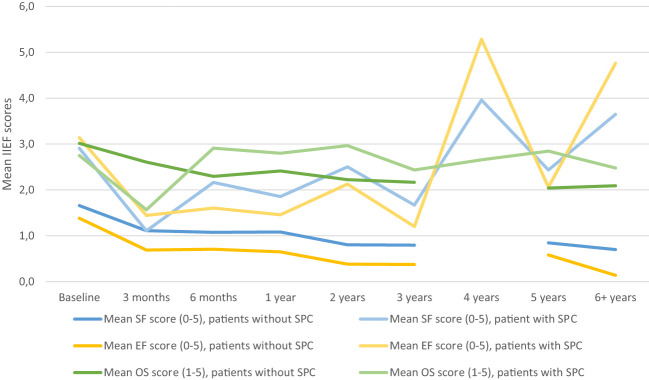
Sexual function (SF mean IIEF-15), erectile function (EF), overall satisfaction (OS) adjusted for sexual preserving cystectomy (SPC). IIEF 5-point Likert scale: 0 = did not attempt intercourse; 1 = never/almost never; 2 = a couple of times; 3 = sometimes; 4 = often; 5 = always/almost always.

In **Figure 4** means of total SF score, OS domain and EF domain were corrected for SPC. The mean OS was higher than the mean SF, whereas the mean EF was lower than the mean SF. To evaluate correlation between QoL and the SF, means of the global QoL of the QLQ-C30 questionnaire were added to the model with means of IIEF-15 questionnaires. There was a positive correlation for QoL and SF (estimate of 0.139, *p*-value=0.004).

### Generalized Linear Mixed Model

In **Supplementary Table 7** the outcomes of the GLMM are depicted per domain of the EORTC-questionnaire, where the impact of SPC, urinary division (Bricker’s ileal conduit or neobladder) and the type of RC (ORC/RARC) were analyzed. Only the type of RC seemed to have an impact on the Global Health status, where patients with a RARC reported a higher Global Health score than those with an ORC (p=0.01). All other domains showed no significant effect regarding the SPC, urinary diversion or RC. In **Supplementary Figures 1**-**15** the unadjusted GLMM outcomes of the domains of the EORTC-QLQC30 questionnaire are shown.

## Discussion

This study showed that sexual function declines significantly after RC and this does not change during six-years of follow-up. The BC patients had, on average, moderate to severe erectile- and sexual dysfunction not only post-RC, but also pre-RC. A SPC and an orthotopic ileal neobladder yielded beneficial effects for the SF. Out of the six patients who received an orthotopic neobladder, four underwent SPC, which likely contributed to better sexual function outcomes. Given the collinearity of age and orthotopic neobladder with SPC, our conclusions emphasize the role of SPC in preserving sexual function post-RC.

The question stands if this statistically significant result is also clinically significant, since there is ED in all groups. A younger age also seemed to increase the SF, although the full effect could not be analyzed. Other studied variables such as smoking habits, type of therapy, comorbidities, ORC vs. RARC, ASA-score, BMI, patient- and tumor status did not have an influence on the SF. This study found a higher mean overall satisfaction (OS) score, and a lower EF score compared to the mean SF score. This shows that although intercourse might not be possible, patients can find satisfaction through other ways of intimacy. Furthermore, a positive correlation was found between QoL and the SF score, which further proves that it should be regarded as an important aspect of patient’s care and follow-up (4).

Consistent with our findings, two recent RCTs revealed that both RARC and ORC had a similar impact on sexual outcomes at 6-month follow-up and 1 year follow-up, with both groups experiencing a significant decline in sexual functioning (21, 22). Additionally, our study revealed that sexual functioning does not change in the long-term follow-up. Besides, in another study, a comparison was made regarding sexual functioning outcomes in patients who underwent a SPC either open or robot-assisted with intracorporeal orthotopic neobladder (23). The results of the study indicated that the RARC group exhibited better sexual function after a one-year follow-up, with 63% of patients experiencing no postoperative erectile dysfunction (IIEF-5 ≥ 22), in contrast to 100% severe ED (IIEF-5 ≤ 7) occurrence in the ORC group. It should be noted that the sample size (11 male patients) in the group was small; these findings support the benefits of RARC over ORC in the context of preserving sexual function when considering the SPC and type of urinary deviation for patients who are candidates for an orthotopic neobladder. These findings may hold importance in discussions with patients regarding their treatment options.

Comparing SF scores, another study reported mean IIEF-5 score in a sexually active population of 22.08 pre-RC and 4.33 post-RC (on a scale 0-25) (14). Although the pre-operative SF is much higher than the results of this study, the post-operative results do correspond with our results. The difference in SF pre-RC is probably a result of the selection of a sexually active population. We chose not to exclude the non-sexually active population because this might have led to an overestimation of the SF. Moreover, we did find that the pre-operative SF was significantly better in the group with a SPC. In a recent study by Abozaid et al., the SF was measured by EORTC-CLC-BLM30, and they noticed an improvement of SF after 12 months post-RC, although it did not reach baseline function, which corresponds to the results of this study (10).

As was to be expected, patients who received a SPC yielded a significant improvement of the SF, compared to those who did not. Part of this difference can be attributed to selection bias; the group with SPC were significantly younger and had a higher preoperative function, which is to be expected since younger, less comorbid men are selected for SPC. It should be noted that although an increase in SF is seen in the figures over the years, further selection bias could overestimate these results since less recurrence might occur compared to the patients who did not receive a SPC. A significant effect between SPC and standard RC was also reported in all previous comparative studies (6, 12–14, 24–26). The study performed by Basiri et al., which also evaluated the IIEF-score, revealed a mean SF of 19.8 in prostate-sparing RC compared to 5.7 in the standard RC (range 0 to 25), these results are comparable to those of this study (25).

Patients with an orthotopic ileal neobladder, preserving body image, had a better SF compared to patients with Bricker’s ileal conduit diversion, although it should be noted that only six patients received an orthotopic ileal neobladder (27). Furthermore, patients who received an orthotopic ileal neobladder were significantly younger than those who received Bricker’s ileal conduit diversion and this group received a SPC more often. It could not be analyzed whether urinary diversion was time-dependent due to the low power of the analysis. The difference in SF between the urinary diversions was also reported in the study by Zippe et al., although it was not clinically significant (14). Another study by Abozaid et al. did not find a significant difference between different types of diversions, although the SF was not assessed with IIEF-15 (10).

However, differences between the elder and younger patients seemed to decrease after RC. The current study reported a significant difference in age between the group with and without SPC. The power of the study was too low to implement age as an interaction term to the model with SPC. It is unknown if the effect of the SPC is dependent by age, nor can it be known from the analysis if age influences SF over time when corrected for SPC. It should be noted that BC is a disease of the elderly and ED is also a result of old age. The prevalence of ED in the Netherlands is 40% in men of 61-70 years old and data from the United States reported ED in 70% in men aged 70 and older (28, 29). As BC is a disease occurring predominantly in older adults, ideally, a control-group would have been added to correct for the ED caused by age, rather than the cancer and its treatments (30).

Contrary to the findings of this study, comorbidities did have a significant influence on the SF or QoL in other studies, especially diabetes mellitus, coronary artery disease and hypertension or multiple comorbidities (11, 13, 31). Type of therapy not having an impact on the SF is an unexpected result, as it is known chemotherapy and radiotherapy do have an influence on both SF and QoL (31, 32). It could be hypothesized that the negative effect of the adjuvant therapy is overshadowed by the massive impact of the RC on the SF.

The QoL outcomes of the EORTC QLQ-C30 questionnaire are comparative to that of other studies (21, 22). The GLMM did not show significant differences in the QoLscores for SPC, UD, or ORC/RARC. Only Health status score was significantly higher in patients with RARC. This significant difference was not seen in other studies (21, 22). Unfortunately, our model could not be analyzed with multiple variables simultaneously due to the small sample size, and thus the full effect could not be analyzed. Another study performed by Mastoianni et al., reported that patients with ORC did experience impairment in role functioning, return to activities and symptoms such as fatigue, dyspnea, insomnia, constipation, diarrhea, financial difficulties and abdominal bloating and flatulence, which was not seen in our study (22).

To our knowledge, this is the first study analyzing SF after RC with a linear mixed model analyses with a long-term follow-up of six years. Despite our study’s valuable findings, we must highlight some important limitations that should be considered when interpreting the results. The relatively small sample size may restrict the generalizability of our findings. Due to this limited sample size, we were unable to include multiple variables in our model simultaneously, which may have led to an underestimation of the relationships among various factors. Additionally, due to the small sample size, a multivariate analysis of variance was not performed. Due to the small data of the individual domains of the IIEF-questionnaire, a linear mixed model could not be performed. Moreover, our study may have been subjected to selection bias, as mentioned earlier. As a result, the characteristics of our sample might not align perfectly with the wider population. Our study’s sample size is consistent with similar research in this field, with previous studies also incorporating relatively small numbers of patients (10, 14, 25). Yet, it is important to recognize that this consistency doesn’t negate the issues associated with smaller sample sizes. As is common in longitudinal studies, we experienced participant attrition and many patients were excluded due to lack of a filled-in baseline questionnaire. The stringent selection criteria we employed resulted in the exclusion of approximately 1677 patients. Thus, while our study offers valuable insights, the effects of these limitations should be carefully considered when interpreting our findings.

For the main outcome of this study, the IIEF-questionnaire, there were missing values, especially for the orgasmic function and intercourse satisfaction domains. It can be hypothesized that for some patients these questions were difficult to answer due to lack of a sexual partner. Rather by taking the sum score of the questions, exploration was performed to reduce underestimation of the SF. Likewise, the exclusion of the non-sexually active population might have led to an overestimation of the SF. By exploration of the questionnaires, over- or underestimation is corrected to the best of our abilities.

While the IIEF-15 questionnaire has specific ED questions that primarily address penetration, other relevant topics such as quality and consistency of erections and loss of penile length are disregarded (2). The etiology of sexuality-related issues is multifactorial and other factors that might influence sexuality and intimacy that were not considered in the present study, may be an impaired physical condition, urinary incontinence, partner issues and psychological symptoms including anxiety, depression, a low self-esteem or a changed self-image (33). These factors could be taken into account in future research.

Penile rehabilitation may have a beneficial effect to increase chances on recovering erectile function in motivated patients. A recent study by Loh-Doyle et al. reported that insufficient pre-operative counselling and absence of partner interest resulted in a lower IIEF-5 score (13). Furthermore, a study by Abozaid et al. showed that penile rehabilitation resulted in a higher SF score (10); penile rehabilitation is not regularly offered for men after RC. But in a one-year prospective study by Hekal et al., it was shown that erectogenic aid increased the SF, especially in patients with SPC (12). Patients in present study did not receive extensive counselling, penile rehabilitation and/or phosphodiesterase inhibitors until problems with sexual functions were indicated by the patient. This may explain a part of the low recovery rates in sexual functions. However, our results suggest that during follow-up, patients find sexual satisfaction through other ways of intimacy even though sexual function scores remain very low. Future studies are necessary to provide more insights in BC patients’ needs and wishes post RC. Furthermore, comparisons should be made between BC patients and other cancer patients, but also the healthy population to determine to what extent the sexual dysfunction can be allocated to the BC and its treatments.

## Conclusions

Post RC, all BC patients have moderate to severe erectile- and sexual function which do not improve during follow up. Factors that lead to a better outcome in sexual functions are nerve sparing surgery (SPC), colinear with the use of an orthotopic ileal neobladder and younger patient age at time of surgery. Nevertheless, sexual function is multifactorial and cannot be allocated to the treatment alone.

## Data availability statement

The raw data supporting the conclusions of this article will be made available by the authors, without undue reservation.

## Ethics statement

The studies involving humans were approved by the Netherlands Cancer Institute – Antoni van Leeuwenhoek Hospital review board number IRBd20-070. The studies were conducted in accordance with the local legislation and institutional requirements. The participants provided their written informed consent to participate in this study.

## Author contributions

Conceptualization: LA, MN, CP. Methodology: CP, LK, MN. Software: CP, LK; Validation: CP, LK, Formal analysis: CP. Investigation: CP, MN, LK, LA, KH. Data Curation: CP. Writing – original draft: CP. Writing – review & editing: CP, MN, LK, KH, LA. Visualization: CP. Supervision: MN, LK, KH, LA. All authors contributed to the article and approved the submitted version.
