# Supplementary material for: Sexual function after radical cystectomy in males with bladder carcinoma: a six-year longitudinal single-centre study
Source: Front Urol. 2023 Oct 3;3:1100516. doi: 10.3389/fruro.2023.1100516 (PMC12327249; doi:10.3389/fruro.2023.1100516)
Supplement: Supplementary file 1 [file DataSheet_1.docx]

# Appendix

**Supplementary table 1: Crude Linear Mixed Model Analysis for mean IIEF-15 score**

| **Estimates of Fixed Effects^a^** | | | | | | | |
| --- | --- | --- | --- | --- | --- | --- | --- |
| Parameter | Estimate | Std. Error | df | t | Sig. | 95% Confidence Interval | |
|  |  |  |  |  |  | Lower Bound | Upper Bound |
| Intercept | 1.404 | .507 | 5.452 | 2.772 | .036 | .134 | 2.674 |
| Baseline | .511 | .518 | 5.963 | .986 | .362 | -.759 | 1.782 |
| 3 months | -.205 | .497 | 5.077 | -.412 | .697 | -1.477 | 1.067 |
| 6 months | -.114 | .508 | 5.492 | -.224 | .831 | -1.385 | 1.157 |
| 1 year | -.215 | .499 | 5.136 | -.431 | .684 | -1.488 | 1.058 |
| 2 years | -.330 | .521 | 6.082 | -.634 | .549 | -1.602 | .941 |
| 3 years | -.475 | .496 | 5.014 | -.959 | .382 | -1.748 | .798 |
| 4 years | -.260 | .504 | 5.421 | -.515 | .627 | -1.527 | 1.007 |
| 5 years | -.275 | .475 | 4.702 | -.579 | .589 | -1.519 | .969 |
| 6+ years | 0^b^ | 0 | . | . | . | . | . |
| a. Dependent Variable: IIEFmean1to15. | | | | | | | |
| b. This parameter is set to zero because it is redundant. | | | | | | | |

**Supplementary table 2: Linear Mixed Model Analysis for mean IIEF-15 score with sexual preservation cystectomy**

| **Estimates of Fixed Effects^a^** | | | | | | | |
| --- | --- | --- | --- | --- | --- | --- | --- |
| Parameter | Estimate | Std. Error | df | t | Sig. | 95% Confidence Interval | |
|  |  |  |  |  |  | Lower Bound | Upper Bound |
| Intercept | 3.648 | .628 | 5.038 | 5.806 | .002 | 2.036 | 5.259 |
| Baseline | -.740 | .671 | 6.476 | -1.103 | .309 | -2.353 | .873 |
| 3 months | -2.540 | .556 | 3.078 | -4.566 | .019 | -4.285 | -.795 |
| 6 months | -1.487 | .638 | 5.172 | -2.331 | .065 | -3.110 | .137 |
| 1 year | -1.794 | .660 | 6.047 | -2.719 | .034 | -3.405 | -.182 |
| 2 years | -1.147 | .732 | 8.478 | -1.567 | .154 | -2.818 | .525 |
| 3 years | -1.980 | .662 | 6.536 | -2.992 | .022 | -3.567 | -.392 |
| 4 years | .312 | .255 | 3.801 | 1.225 | .291 | -.410 | 1.033 |
| 5 years | -1.213 | .732 | 12.848 | -1.658 | .121 | -2.796 | .369 |
| 6+ years | 0^b^ | 0 | . | . | . | . | . |
| No sexual preservation (SP) | -2.949 | .679 | 4.914 | -4.340 | .008 | -4.705 | -1.193 |
| Sexual preservation (SP) | 0^b^ | 0 | . | . | . | . | . |
| Baseline * No SP | 1.699 | .731 | 6.483 | 2.325 | .056 | -.057 | 3.455 |
| Baseline * SP | 0^b^ | 0 | . | . | . | . | . |
| 3 months * No SP | 2.952 | .605 | 3.077 | 4.877 | .016 | 1.053 | 4.851 |
| 3 monhts * SP | 0^b^ | 0 | . | . | . | . | . |
| 6 months * No SP | 1.862 | .689 | 4.983 | 2.705 | .043 | .091 | 3.634 |
| 6 months * SP | 0^b^ | 0 | . | . | . | . | . |
| 1 year * No SP | 2.176 | .703 | 5.567 | 3.096 | .023 | .423 | 3.929 |
| 1 year * SP | 0^b^ | 0 | . | . | . | . | . |
| 2 years * No SP | 1.251 | .784 | 7.938 | 1.595 | .150 | -.560 | 3.061 |
| 2 years * SP | 0^b^ | 0 | . | . | . | . | . |
| 3 years * No SP | 2.078 | .703 | 6.058 | 2.956 | .025 | .362 | 3.794 |
| 3 years * SP | 0^b^ | 0 | . | . | . | . | . |
| 4 years * No SP | 0^b^ | 0 | . | . | . | . | . |
| 5 years * No SP | 1.360 | .765 | 11.501 | 1.779 | .102 | -.314 | 3.035 |
| 5 years * SP | 0^b^ | 0 | . | . | . | . | . |
| 6 years * No SP | 0^b^ | 0 | . | . | . | . | . |
| 6 years * SP | 0^b^ | 0 | . | . | . | . | . |
| a. Dependent Variable: IIEFmean1to15. | | | | | | | |
| b. This parameter is set to zero because it is redundant. | | | | | | | |

**Supplementary table 3: Linear Mixed Model Analysis for mean IIEF-15 score with sexual preservation cystectomy and urinary diversion**

| **Estimates of Fixed Effects^a^** | | | | | | | |
| --- | --- | --- | --- | --- | --- | --- | --- |
| Parameter | Estimate | Std. Error | df | t | Sig. | 95% Confidence Interval | |
|  |  |  |  |  |  | Lower Bound | Upper Bound |
| Intercept | 4,395440 | ,655109 | 5,348 | 6,709 | ,001 | 2,743840 | 6,047039 |
| Baseline | -,615470 | ,666726 | 5,587 | -,923 | ,394 | -2,276537 | 1,045598 |
| 3 months | -2,409832 | ,562743 | 2,773 | -4,282 | ,027 | -4,286427 | -,533237 |
| 6 months | -1,393908 | ,636222 | 4,461 | -2,191 | ,087 | -3,090664 | ,302848 |
| 1 year | -1,714239 | ,654100 | 5,065 | -2,621 | ,046 | -3,389152 | -,039326 |
| 2 years | -,988335 | ,730692 | 7,450 | -1,353 | ,216 | -2,695219 | ,718549 |
| 3 years | -1,876426 | ,654597 | 5,596 | -2,867 | ,031 | -3,506599 | -,246252 |
| 4 years | ,306195 | ,251961 | 3,208 | 1,215 | ,306 | -,467012 | 1,079402 |
| 5 years | -1,089822 | ,710555 | 12,098 | -1,534 | ,151 | -2,636605 | ,456960 |
| 6+ years | 0^b^ | 0 | . | . | . | . | . |
| No sexual preservation (SP) | -2,442890 | ,682650 | 4,366 | -3,579 | ,020 | -4,277131 | -,608649 |
| Sexual preservation (SP) | 0^b^ | 0 | . | . | . | . | . |
| Baseline * No SP | 1,517030 | ,725328 | 5,594 | 2,092 | ,085 | -,289454 | 3,323513 |
| Baseline * SP | 0^b^ | 0 | . | . | . | . | . |
| 3 months * No SP | 2,806672 | ,610804 | 2,754 | 4,595 | ,023 | ,760699 | 4,852645 |
| 3 monhts * SP | 0^b^ | 0 | . | . | . | . | . |
| 6 months * No SP | 1,737278 | ,685828 | 4,286 | 2,533 | ,060 | -,117807 | 3,592363 |
| 6 months * SP | 0^b^ | 0 | . | . | . | . | . |
| 1 year * No SP | 2,118963 | ,696475 | 4,660 | 3,042 | ,031 | ,288662 | 3,949264 |
| 1 year * SP | 0^b^ | 0 | . | . | . | . | . |
| 2 years * No SP | 1,080429 | ,783063 | 6,997 | 1,380 | ,210 | -,771355 | 2,932214 |
| 2 years * SP | 0^b^ | 0 | . | . | . | . | . |
| 3 years * No SP | 1,972888 | ,695257 | 5,207 | 2,838 | ,035 | ,206788 | 3,738989 |
| 3 years * SP | 0^b^ | 0 | . | . | . | . | . |
| 4 years * No SP | 0^b^ | 0 | . | . | . | . | . |
| 5 years * No SP | 1,230202 | ,744395 | 10,641 | 1,653 | ,128 | -,414979 | 2,875383 |
| 5 years * SP | 0^b^ | 0 | . | . | . | . | . |
| 6 years * No SP | 0^b^ | 0 | . | . | . | . | . |
| 6 years * SP | 0^b^ | 0 | . | . | . | . | . |
| Bricker’s Ileal Conduit | -1,308705 | ,373074 | 49,828 | -3,508 | ,001 | -2,058110 | -,559299 |
| Orthotopic Ileal Neobladder | 0^b^ | 0 | . | . | . | . | . |
| a. Dependent Variable: IIEFmean1to15. | | | | | | | |
| b. This parameter is set to zero because it is redundant. | | | | | | | |

**Supplementary table 4: Linear Mixed Model Analysis for mean Erectile Function score with sexual preservation cystectomy**

| **Estimates of Fixed Effects^a^** | | | | | | | |
| --- | --- | --- | --- | --- | --- | --- | --- |
| Parameter | Estimate | Std. Error | df | t | Sig. | 95% Confidence Interval | |
|  |  |  |  |  |  | Lower Bound | Upper Bound |
| Intercept | 4,760112 | ,774210 | 5,764 | 6,148 | ,001 | 2,846765 | 6,673459 |
| Baseline | -1,621529 | ,823818 | 7,284 | -1,968 | ,088 | -3,554248 | ,311190 |
| 3 months | -3,315362 | ,700232 | 3,819 | -4,735 | ,010 | -5,296389 | -1,334335 |
| 6 months | -3,154991 | ,778614 | 5,719 | -4,052 | ,007 | -5,083085 | -1,226897 |
| 1 year | -3,303542 | ,800326 | 6,561 | -4,128 | ,005 | -5,222011 | -1,385074 |
| 2 years | -2,631815 | ,881158 | 8,773 | -2,987 | ,016 | -4,633010 | -,630620 |
| 3 years | -3,559322 | ,798937 | 6,581 | -4,455 | ,003 | -5,473192 | -1,645452 |
| 4 years | ,526184 | ,347707 | 6,377 | 1,513 | ,178 | -,312566 | 1,364934 |
| 5 years | -2,699759 | ,922583 | 10,583 | -2,926 | ,014 | -4,740151 | -,659367 |
| 6+ years | 0^b^ | 0 | . | . | . | . | . |
| No sexual preservation (SP) | -4,622115 | ,841259 | 5,669 | -5,494 | ,002 | -6,710111 | -2,534119 |
| Sexual preservation (SP) | 0^b^ | 0 | . | . | . | . | . |
| Baseline * No SP | 2,866404 | ,900510 | 7,332 | 3,183 | ,015 | ,756420 | 4,976389 |
| Baseline * SP | 0^b^ | 0 | . | . | . | . | . |
| 3 months * No SP | 3,863858 | ,764703 | 3,831 | 5,053 | ,008 | 1,703200 | 6,024517 |
| 3 monhts * SP | 0^b^ | 0 | . | . | . | . | . |
| 6 months * No SP | 3,724991 | ,844707 | 5,576 | 4,410 | ,005 | 1,619379 | 5,830602 |
| 6 months * SP | 0^b^ | 0 | . | . | . | . | . |
| 1 year * No SP | 3,816268 | ,858646 | 6,144 | 4,445 | ,004 | 1,727149 | 5,905388 |
| 1 year * SP | 0^b^ | 0 | . | . | . | . | . |
| 2 years * No SP | 2,873292 | ,948877 | 8,314 | 3,028 | ,016 | ,699499 | 5,047085 |
| 2 years * SP | 0^b^ | 0 | . | . | . | . | . |
| 3 years * No SP | 3,793853 | ,854657 | 6,133 | 4,439 | ,004 | 1,713505 | 5,874201 |
| 3 years * SP | 0^b^ | 0 | . | . | . | . | . |
| 4 years * No SP | 0^b^ | 0 | . | . | . | . | . |
| 5 years * No SP | 3,143506 | ,975534 | 9,919 | 3,222 | ,009 | ,967471 | 5,319541 |
| 5 years * SP | 0^b^ | 0 | . | . | . | . | . |
| 6 years * No SP | 0^b^ | 0 | . | . | . | . | . |
| 6 years * SP | 0^b^ | 0 | . | . | . | . | . |
| a. Dependent Variable: IIEFmeanEF. | | | | | | | |
| b. This parameter is set to zero because it is redundant. | | | | | | | |

**Supplementary table 5: Linear Mixed Model Analysis for mean Overall Satisfaction score with sexual preservation cystectomy**

| **Estimates of Fixed Effects^a^** | | | | | | | |
| --- | --- | --- | --- | --- | --- | --- | --- |
| Parameter | Estimate | Std. Error | df | t | Sig. | 95% Confidence Interval | |
|  |  |  |  |  |  | Lower Bound | Upper Bound |
| Intercept | 2,479231 | ,748419 | 14,928 | 3,313 | ,005 | ,883340 | 4,075122 |
| Baseline | ,269006 | ,755102 | 15,284 | ,356 | ,727 | -1,337860 | 1,875871 |
| 3 months | -,912052 | ,616892 | 6,385 | -1,478 | ,187 | -2,399737 | ,575632 |
| 6 months | ,431182 | ,733157 | 13,015 | ,588 | ,567 | -1,152526 | 2,014890 |
| 1 year | ,319835 | ,915637 | 29,546 | ,349 | ,729 | -1,551352 | 2,191022 |
| 2 years | ,487449 | ,780838 | 17,589 | ,624 | ,540 | -1,155787 | 2,130685 |
| 3 years | -,043064 | ,845530 | 22,083 | -,051 | ,960 | -1,796205 | 1,710077 |
| 4 years | ,176880 | ,240732 | 4,123 | ,735 | ,502 | -,483692 | ,837452 |
| 5 years | ,368346 | 1,216677 | 13,801 | ,303 | ,767 | -2,244708 | 2,981400 |
| 6+ years | 0^b^ | 0 | . | . | . | . | . |
| No sexual preservation (SP) | -,389435 | ,805545 | 14,322 | -,483 | ,636 | -2,113518 | 1,334649 |
| Sexual preservation (SP) | 0^b^ | 0 | . | . | . | . | . |
| Baseline * No SP | ,656233 | ,816253 | 14,884 | ,804 | ,434 | -1,084751 | 2,397217 |
| Baseline * SP | 0^b^ | 0 | . | . | . | . | . |
| 3 months * No SP | 1,427093 | ,667746 | 6,313 | 2,137 | ,074 | -,187401 | 3,041587 |
| 3 monhts * SP | 0^b^ | 0 | . | . | . | . | . |
| 6 months * No SP | -,225659 | ,781689 | 12,199 | -,289 | ,778 | -1,925733 | 1,474416 |
| 6 months * SP | 0^b^ | 0 | . | . | . | . | . |
| 1 year * No SP | ,002094 | ,977091 | 27,119 | ,002 | ,998 | -2,002319 | 2,006506 |
| 1 year * SP | 0^b^ | 0 | . | . | . | . | . |
| 2 years * No SP | -,355384 | ,818333 | 15,381 | -,434 | ,670 | -2,095865 | 1,385097 |
| 2 years * SP | 0^b^ | 0 | . | . | . | . | . |
| 3 years * No SP | ,119475 | ,895589 | 19,880 | ,133 | ,895 | -1,749412 | 1,988362 |
| 3 years * SP | 0^b^ | 0 | . | . | . | . | . |
| 4 years * No SP | 0^b^ | 0 | . | . | . | . | . |
| 5 years * No SP | -,416942 | 1,289789 | 12,716 | -,323 | ,752 | -3,209707 | 2,375824 |
| 5 years * SP | 0^b^ | 0 | . | . | . | . | . |
| 6 years * No SP | 0^b^ | 0 | . | . | . | . | . |
| 6 years * SP | 0^b^ | 0 | . | . | . | . | . |
| a. Dependent Variable: IIEFmeanOS. | | | | | | | |
| b. This parameter is set to zero because it is redundant. | | | | | | | |

**Supplementary table 6: -2 Log Likelihood and number of parameters for linear mixed models**

| Linear Mixed Model | -2 Log Likelihood | Number of parameters |
| --- | --- | --- |
| Crude sexual function (mean IIEF-15) | 541.92 | 20 |
| Sexual function (mean IIEF-15) with SPC | 508.44 | 28 |
| Sexual function (mean IIEF-15) with SPC and urinary diversion | 484.81 | 29 |
| Erectile function with SPC | 540.76 | 28 |
| Overall satisfaction with SPC | 593.40 | 28 |

**Supplementary table 7**: The domains of the EORTC-QLQC30 questionnaire tested for sexual preserving cystectomy, urinary division and open- or robot-assisted radical cystectomy using Generalized Linear Mixed Model.

| **Time point** | **Coefficient SPC (SE)** | ***p*-value SPC** | **Coefficient UD (SE)** | ***p*-value UD** | **Coefficient ORC/RARC (SE)** | ***p*-value ORC/RARC** |
| --- | --- | --- | --- | --- | --- | --- |
| Global Health Status | -0.03 (0.06) | 0.96 | 0.05 (0.08) | 0.56 | 0.12 (0.05) | 0.01 |
| Physical functioning | -0.05 (0.70) | 0.49 | -0.08 (0.09) | 0.41 | 0.06 (0.06) | 0.26 |
| Role functioning | 0.02 (0.08) | 0.82 | -0.04 (0.11) | 0.97 | 0.08 (0.65) | 0.20 |
| Emotional functioning | 0.07 (0.06) | 0.25 | 0.14 (0.08) | 0.07 | -0.01 (-0.05) | 0.81 |
| Cognitive functioning | 0.03 (0.47) | 0.95 | 0.03 (0.06) | 0.63 | 0.02 (0.04) | 0.59 |
| Social functioning | 0.03 (0.11) | 0.81 | -0.08 (0.14) | 0.55 | -0.06 (0.08) | 0.45 |
| Fatigue | -0.02 (0.16) | 0.91 | 0.06 (0.20) | 0.98 | -0.10 (0.12) | 0.38 |
| Nausea and vomiting | -0.43 (0.41) | 0.30 | 0.05 (0.59) | 0.94 | 0.07 (0.29) | 0.82 |
| Pain | 0.03 (0.22) | 0.89 | 0.02 (0.25) | 0.94 | -0.21 (0.16) | 0.20 |
| Dyspnea | 0.19 (0.15) | 0.19 | 0.09 (0.16) | 0.58 | -0.07 (0.11) | 0.51 |
| Insomnia | 0.07 (0.14) | 0.64 | 0.09 (0.15) | 0.58 | -0.17 (0.10) | 0.12 |
| Appetite loss | 0.09 (0.28) | 0.98 | 0.27 (0.34) | 0.43 | 0.07 (0.20) | 0.74 |
| Constipation | 0.31 (0.22) | 0.16 | 0.30 (0.22) | 0.18 | -0.03 (0.12) | 0.82 |
| Diarrhea | 0.07 (0.12) | 0.55 | -0.01 (0.15) | 0.99 | 0.03 (0.08) | 0.74 |
| Financial difficulties | -0.03 (0.12) | 0.80 | -0.02 (0.14) | 0.99 | -0.14 (0.09) | 0.13 |

ORC = Open Radical Cystectomy (coded as 2). RARC = Robot-Assisted Radical Cystectomy (coded as 1). SE = Standard Error. SPC = Sexual Preserving Cystectomy (no SPC coded as 1, SPC coded as 2). UD = Urinary Diversion (Bricker coded as 1, Neobladder defined as 2, Indiana pouch left out due to small sample size).

**Supplementary figure 1**: Global health status score (0-100) from the EORTC QLQ-C30 questionnaire

**Supplementary figure 2**: Physical functioning score (0-100) from the EORTC QLQ-C30 questionnaire

**Supplementary figure 3**: Role functioning score (0-100) from the EORTC QLQ-C30 questionnaire

**Supplementary figure 4**: Emotional functioning score (0-100) from the EORTC QLQ-C30 questionnaire

**Supplementary figure 5**: Cognitive functioning score (0-100) from the EORTC QLQ-C30 questionnaire

**Supplementary figure 6**: Social functioning score (0-100) from the EORTC QLQ-C30 questionnaire

**Supplementary figure 7**: Fatigue score (0-100) from the EORTC QLQ-C30 questionnaire

**Supplementary figure 8**: Nausea and vomiting score (0-100) from the EORTC QLQ-C30 questionnaire

**Supplementary figure 9**: Pain score (0-100) from the EORTC QLQ-C30 questionnaire

**Supplementary figure 10**: Dyspnea score (0-100) from the EORTC QLQ-C30 questionnaire

**Supplementary figure 11**: Insomnia score (0-100) from the EORTC QLQ-C30 questionnaire

**Supplementary figure 12**: Appetite loss score (0-100) from the EORTC QLQ-C30 questionnaire

**Supplementary figure 13**: Constipation score (0-100) from the EORTC QLQ-C30 questionnaire

**Supplementary figure 14**: Diarrhea score (0-100) from the EORTC QLQ-C30 questionnaire

**Supplementary figure 15**: Financial difficulties (0-100) from the EORTC QLQ-C30 questionnaire
